# Editorial: Actinobacteria: Prolific Producers of Bioactive Metabolites

**DOI:** 10.3389/fmicb.2020.01612

**Published:** 2020-08-21

**Authors:** Learn-Han Lee, Bey-Hing Goh, Kok-Gan Chan

**Affiliations:** ^1^Novel Bacteria and Drug Discovery Research Group, Microbiome and Bioresource Research Strength, Jeffrey Cheah School of Medicine and Health Sciences, Monash University Malaysia, Subang Jaya, Malaysia; ^2^College of Pharmaceutical Sciences, Zhejiang University, Hangzhou, China; ^3^Biofunctional Molecule Exploratory Research Group (BMEX), School of Pharmacy, Monash University Malaysia, Bandar Sunway, Malaysia; ^4^Division of Genetics and Molecular Biology, Institute of Biological Sciences, Faculty of Science, University of Malaya, Kuala Lumpur, Malaysia; ^5^International Genome Centre, Jiangsu University, Zhenjiang, China

**Keywords:** Actinobacteria, prolific producers, Streptomyces, bioactive metabolites, bioprospecting

## Introduction

For decades, scientists have conducted bioprospecting on Actinobacteria for the discovery of novel genera producing bioactive metabolites (Atalan et al., 2000; Bull et al., 2000; Goodfellow et al., 2018). Actinobacteria are the most prolific source of bioactive secondary metabolites, with diverse structural complexity (Takahashi, 2004). Actinobacteria*-*derived metabolites exhibit a wide spectrum of bioactivities, including antimicrobial (Umezawa et al., 1966), antifungal (Fukuda et al., 2005), anticancer (Omura et al., 1977), antiparasitic (Ǒmura, 2003), and immunosuppressive activities (Barka et al., 2016). Thus, Actinobacteria continue to fuel biotechnology and medicine sectors with new biomolecules. In this Research Topic, a total of nine articles were published, illustrating wide arrays of bioactive metabolites produced by Actinobacteria derived from diverse ecosystems and the biosynthetic regulatory mechanisms of these metabolites.

## *Streptomyces—*a Powerhouse of Secondary Metabolites

After decades of bioprospecting, *Streptomyces* remains a priority due to its unsurpassed competency in producing a stunning multitude of diversified bioactive metabolites (Fiedler and Goodfellow, 2004; Goodfellow and Fiedler, 2010). The ability of *Streptomyces* to provide sources for new antibiotics against methicillin-resistant *Staphylococcus aureus* (MRSA) was highlighted (Kemung et al.). For instance, griseusin A, marinopyrrole A, and polyketomycin are several potent anti-MRSA compounds produced by *Streptomyces*, showing great promise for future clinical use. Balasubramanian et al. reported a compound (SKC_3_) from a marine sponge-derived *Streptomyces* sp. SBT_34_8 extract with antagonistic effects against growth and biofilm formation of several staphylococcal strains. Yu et al. reported two new fatty acids with nitrile group Borrelidins J and K, produced by *Streptomyces rochei* MB037, exhibiting strong activities against *S. aureus*. Besides *Streptomyces*, other genera such as *Microbacterium* within the phylum Actinobacteria derived from marine sponges also showed promising antibacterial activity against MRSA (Santos et al.).

Microbial secondary metabolites exhibited many useful applications for humankind. Many antibiotics derived from *Streptomyces* act as a defense mechanism to mediate competitive interspecies interactions (Chevrette et al., 2019). Tenconi et al. demonstrated the production of broad-spectrum molecules, the prodiginines associated with programmed cell death of the host, *S. coelicolor*. Hence, researchers suggested that the third use for antibiotics would be as molecules for self-toxicity to regulate cell proliferation other than serving as traditionally perceived tools for inter- or intra-species communication (Mccormick and Flärdh, 2012).

## Untapped Reservoir of Biodiversity for Bioprospecting

The widespread occurrences of drug resistance in cancer and pathogens have rendered many medicines ineffective, and new strategies are therefore needed to uncover new agents (Antoraz et al., 2015). Exploring new taxa from untapped sources is an efficient strategy in searching for new drug leads/chemical scaffolds, as taxonomic diversity correlates to chemical diversity (Harvey, 2000; Sayed et al., 2020). Untapped environments like deep oceans (Abdel-Mageed et al., 2010) and mangroves (Hong et al., 2009) are proven a prolific source of bioactive Actinobacteria (Bull et al., 2005; Bull and Goodfellow, 2019). Moreover, Rangseekaew and Pathom-aree summarized that cave ecosystems harbor novel and diverse Actinobacteria, with promising bioactive metabolites, with a total of 47 species within 30 genera, including seven types of novel genera of Actinobacteria reported between 1999 and 2018. The coastal salt marsh plants represent another reservoir for diverse and novel endophytic Actinobacteria with promising biosynthetic capabilities as biocontrol agents and fibrinolytic enzymes (Chen et al.).

## Co-Cultivation-, Genome-, and Modern Metabolomics-Based Bioprospecting Approaches in Actinobacteria

Actinobacteria have widely differing genome sizes, ranging from 1 to 12 Mb (Větrovský and Baldrian, 2013), where biologically active compounds are genetically encoded as biosynthetic gene clusters (BGCs). The advancement in Next-Generation Sequencing (NGS) technologies enhanced the understanding of secondary metabolite biosynthesis potentials of Actinobacteria (Nouioui et al., 2018). The genomic analysis revealed Actinobacteria capable of producing many more compounds than were observed in *in vitro* culture, indicating many of these BGCs are silent or weakly expressed under standard laboratory conditions.

Given that microbes commonly coexist in diverse communities in nature, microbes interact with each other *via* production of potentially useful bioactive secondary metabolites. Co-cultivation is an effective approach to simulate authentic circumstances in the environment. This approach has been shown to activate the silent genes and increase the yield of useful compounds by culturing two or more microorganisms in the same environment (Rateb et al., 2013). Herein, Yu et al. demonstrated co-culture of *Streptomyces rochei* MB037 with the gorgonian-derived fungus *Rhinocladiella similis* 35, which led to the isolation of three novel antibacterial compounds.

The ability to unravel the whole genomic sequences of *Streptomyces* strains has enabled the pleiotropic regulation or effective manipulation of regulatory genes in pathway-specific *Streptomyces* (Van Der Heul et al., 2018). Hou et al. revealed a potential role of a novel regulatory family, LmbU, to be used for yield enhancement of lincomycin from *Streptomyces*. Clearly, the enhancement of our understanding on the regulation of specialized metabolic gene clusters is the key to yielding improvement of a target compound for large-scale manufacturing in Actinobacteria. Despite the advances of bioinformatic prediction tools, new genomes are increasingly becoming available for identification in newly isolated microbial strains. Analytical chemistry techniques are indispensable in uncovering the full biosynthetic potential of microbes with use of hyphenated techniques, particularly high-resolution mass spectrometry (MS) and nuclear magnetic resonance spectroscopy (NMR) for systematic assessment of novel molecules (Goodfellow and Fiedler, 2010). Schneider et al. identified a new analog of geninthiocin, a thiopeptide antibiotic, named as geninthiocin B, and its BGCs from a *Streptomyces* sp. derived from a lichen *Lepidostroma yunnana* sp. nov. sample via genome mining coupled with MS- and NMR-based metabolomic approaches.

## Conclusion

In summary, this Research Topic enhances our knowledge on the immense biological potential of Actinobacteria. The fact that this bacteria can easily be found in diverse ecosystems, including caves, coastal marshes, and marine sponges, further signifies the irreplaceable role of Actinobacteria in the field of biotechnology and medicine. Furthermore, the need for the development of new and effective bioprospecting tools is important in expediting the discovery process of potentially novel compounds from these biologically active Actinobacteria. Only the combination of technologies between microbiology, molecular biology, and analytical chemistry will continue to uncover the vast hidden scaffolds for novel bioactive secondary metabolites produced by Actinobacteria.

## Author Contributions

L-HL, B-HG, and K-GC contributed to the literature review and writing of the project. The project was founded by L-HL. All authors contributed to the article and approved the submitted version.

## Conflict of Interest

The authors declare that the research was conducted in the absence of any commercial or financial relationships that could be construed as a potential conflict of interest.
